# Corrosion Behavior of Aluminium-Coated Cans

**DOI:** 10.3390/ma16031041

**Published:** 2023-01-24

**Authors:** Mohammed Almoiqli, Khalid N. Alharbi, Maha Abdallah Alnuwaiser, Ghaidaa Yajizi, Shoug Alshoshan, Wed Baduways, Muntathir I. Albeladi, Rashed S. Alsanea, Talal A. Aljohani

**Affiliations:** 1 Nuclear Science Research Institute, King Abdulaziz City for Science and Technology, Riyadh 11442, Saudi Arabia; 2 Department of Chemistry, College of Science, Princess Nourah Bint Abdulrahman University, P.O. Box 84428, Riyadh 11671, Saudi Arabia; 3 Materials Science Research Institute, King Abdulaziz City for Science and Technology, Riyadh 11442, Saudi Arabia

**Keywords:** aluminum cans, corrosion, polarization, coating

## Abstract

Hundreds of billions of aluminium-based cans are manufactured and used every year worldwide including those containing soft drinks. This study investigates and evaluates the performance and quality of two well-known energy and soft drinks brands, Green Cola and Red Bull. Recent health hazards and concerns have been associated with aluminium leakage and bisphenol A (BPA) dissociation from the can’s internal protective coating. The cans were examined under four conditions, including coated and uncoated samples, the soft drink’s main solution, and 0.1 M acetic acid solution. Electrochemical measurements such as potentiodynamic polarization and impedance spectroscopy (EIS), element analyses using inductively coupled plasma optical emission spectrometry (ICP-OES), and energy dispersive X-ray spectroscopy (EDS) were performed. In addition, sample characterization by scanning electron microscopy (SEM) and X-ray diffraction spectroscopy (XRD) were employed to comprehensively study and analyze the effect of corrosion on the samples. Even though the internal coating provided superior corrosion protection concerning main or acetic acid solutions, it failed to prevent aluminium from dissolving in the electrolyte. Green Cola’s primary solution appears to be extremely corrosive, as the corrosion rate increased by approximately 333% relative to the acetic acid solution. Uncoated samples resulted in increases in the percentage of oxygen, the appearance of more corrosion spots, and decreases in crystallinity. The ICP-OES test detected dangerous levels of aluminium in the Green Cola solution, which increased significantly after increasing the conductivity of the solution.

## 1. Introduction

Aluminium (Al) is a common industrial metal, especially in beverage and food packaging. The inclusion of more electropositive components in soft drinks, such as chloride and copper ions, facilitates the corrosion of aluminium and its alloys [1]. Although aluminium is a relatively corrosion-impervious metal, drinks with a low pH can dissolve the aluminium oxide layer, which serves as a natural passivation layer. Since the invention of the metal can more than two centuries ago, the globe produces more than 250 billion of them annually [2]. Leaking metal from cans into drinks can cause Alzheimer’s, Parkinson’s, and multiple sclerosis. Aluminium food and drink containers have a thin polymer coating or internal coating layer to reduce metal-to-product contact and corrosion. Thin-layer corrosion protection includes vinylic or phenolic lacquers and epoxy resins [3]. Marijan Seruga and Damir Hasenay say aluminium leaching into soft drinks is a slow, time-dependent process influenced by PH level and brand [4]. Oxygen concentration in the package; pH; product composition; dissolved salts, ions, and molecules; and the environment, temperature, and pressure all affect metal corrosion. Depolarizing oxygen and dissolved ions accelerate corrosion [5].

Epoxy resins are used in advanced polymer materials because of their thermal, mechanical, and corrosion resistance [6]. Epoxies are thermoset polymers that form strong bonds. Epoxy in the can’s interior coating keeps canned goods fresh. This type of material must be inert enough not to harm human health or food quality [7]. Several studies found that epichlorohydrin and bisphenol A (BPA) can leach into food or drinks during the epoxies’ reaction [8]. Numerous studies have been published on the biological impacts and health concerns of bisphenol A (BPA). BPA exposure has been linked to reproductive system abnormalities, cardiovascular illness, and nervous system development. [9]. This affects kids’ behaviour. New research links BPA to hypertension, type 2 diabetes, and cardiovascular disease [10].

Electrochemical and impedance spectroscopy methods are widely used to investigate coating layers in metallic organic coating [11,12] and to assess the barrier properties and corrosion behavior of coated cans [13]. Using the impedance technique, Esteves et al. found one time constant and decreased corrosion resistance for guarana and tonic water. However, for cola soft drinks, two time constants were observed. The explanation of observing two time constants was a more aggressive solution that caused a coating failure [12]. This study examined the internal coatings of Red Bull and Green Cola cans using open-circuit potential measurements, electrochemical impedance spectroscopy, polarization curves, scanning electron microscopy, X-ray diffraction spectroscopy, and elemental analysis using inductively coupled plasma optical emission spectrometry (ICP-OES). These techniques studied can lower corrosion and aluminium leakage.

## 2. Materials and Methods

### 2.1. Characterization

SEM (JEOL JSM-IT300LV, JEOL, Tokyo, Japan) with 100×, 250×, 500× and 1000× magnification) was used to examine aluminium alloy samples before and after corrosion. ICP-OES (inductively coupled plasma-optical emission spectrometry), an analytical technique in which the concentration of elements in (mostly water-dissolved) samples could be determined, was employed for Green Cola and Red Bull solutions.

### 2.2. Samples

Initially, a 1 cm^2^ sheet cut from both Geen Cola and Red Bull cans. For the coated samples, the 1 cm^2^ directly transferred to the corrosion electrochemical cell without any further preparation. However, for the uncoated samples, the inner coating was removed by polishing the samples with SiC paper 1200. It should be noted that the exposed area of the working electrode is 0.785 cm^2^. Samples were examined in terms of two types of corrosion electrolytes; namely the main solution for the drink (MS) or in acetic acid (Ac-OH). The samples were named as follows:

MS-WC denotes samples examined in the main solution with the presence of an internal coating (WC).

MS-WOC denotes samples examined in the main solution without the presence of an internal coating (WOC). The same procedures were applied to acetic acid. 

The content of Geen Cola as labeled on the can is as follows:

A Hellas S.A., Greece, 14564 Green Cola bottle containing: water, carbon dioxide, colourant: sulphite, ammonia and caramel, acidifiers: tartaric acid and malic acid, sweeteners: steviol glycosides (less than 80 mg/L) and sucralose (less than 200 mg/L), natural flavourings, natural caffeine: 10.5 mg/100 mL, and salt: 0.03 g/100 mL. 

The content of Red Bull as labeled on the can is as follows: a Red Bull beverage can produced in Switzerland, filled by Rauch Trading AG, Widnau, Switzerland for Red Bull GmbH, Fuschl am See, Austria. Its nutrition information per 100 mL contains the following: water, sugar, glucose, citric acid, carbon dioxide, taurine (400 mg/100 mL), acidity regulator sodium bicarbonate, natural and artificial flavours (vanilla, pineapple, berries), acidity regulator magnesium carbonate, colour caramel, caffeine (32 mg/100 mL), niacin, pantothenic acid, vitamin B6, colour riboflavin, vitamin B12. 

Furthermore, 1 M KCl as a supporting electrolyte was added to all tested solutions to enhance the solution conductivity.

### 2.3. Electrochemical Experiments

Electrochemical experiments were performed using a traditional three-electrode flat cell containing a platinum mesh as a counter electrode, an Ag/AgCl/KClsat as a reference electrode, and a working electrode with the surface of 0.785 cm^2^. An SP-200 potentiostat (BioLogic Company, Grenoble, France) was employed to run four electrochemical experiments at room temperature using EC Laboratory software for data fitting. Initially, an open circuit potential was measured for 1 h. Subsequently, electrochemical impedance spectroscopy measurements were obtained using an AC signal at an open-circuit potential in the 10^−1^ to 10^5^ Hz frequency range by the application of a sinusoidal voltage at ±10 mV. Additionally, double-layer capacitance (Q), F.s^(n−1)^.cm^−2^, was calculated using the following formula [14]:

(1) $$Q=Y_{0}{\left({ꙍ}_{m}\right)}^{n-1}\;$$

*Y*_0_ is a proportional factor, ꙍ_m_ is the angular frequency at the maximum impedance of the imaginary axis, and n is a deviation factor from −1 to +1 from a pure inductor to a pure capacitor. Then, potentiodynamic polarization was performed at a scan rate of 0.166 mV/s and different working electrode potentials of +500 mV and −250 mV with respect to OCP. 

The corrosion current density i_corr_ is calculated using the following formula [15]:

(2) $$\mathrm{icorr}=i\{\exp\;\left(\frac{2.303\left(E-E_{\mathrm{corr}}\right)}{b_{a}}\right)-\exp\left(-\frac{2.303\;\left(E-E_{\mathrm{corr}}\right)}{b_{c}}\right)\}\;\;$$

where i, E, E_corr_, b_c_, and b_a_ are the current density, applied potential, corrosion potential, cathodic Tafel slope, and anodic Tafel slope, respectively, which are calculated using EC Laboratory software for data fitting. 

The corrosion rate (*r_corr_* in mils per year, mpy), was calculated based on the following [16]:

(3) $$r_{corr}=\frac{0.129\times MW\times icorr}{\rho \times n}$$

where *i_corr_*, MW, ρ, and n represent the corrosion current density (µA cm^−2^), atomic mass of aluminium alloy 3004 (g/mol), sample density (g/cm^3^), and number of electrons exchanged by the corrosion reaction, respectively. Finally, a cyclic polarization scan from +500 mV to −250 mV with respect to OCP at a scan rate of 1 mV/s was performed for hysteresis analyses and examination of localized corrosion susceptibility.

## 3. Results

### 3.1. Electrochemical Measurements

#### 3.1.1. Open Circuit Potential

**Figure 1:**
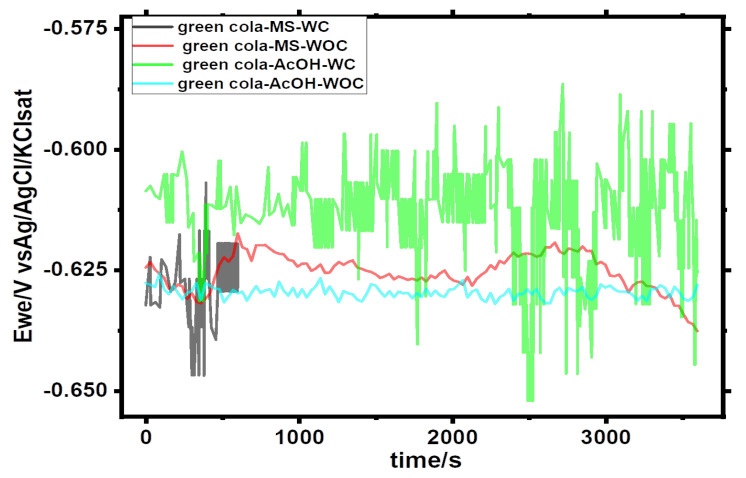
Open circuit potential of Green Cola samples with (WC) and without (WOC) an organic coating in main (MS) and acetic acid (AcOH) solutions; Ewe: working electrode potential.

**Figure 2:**
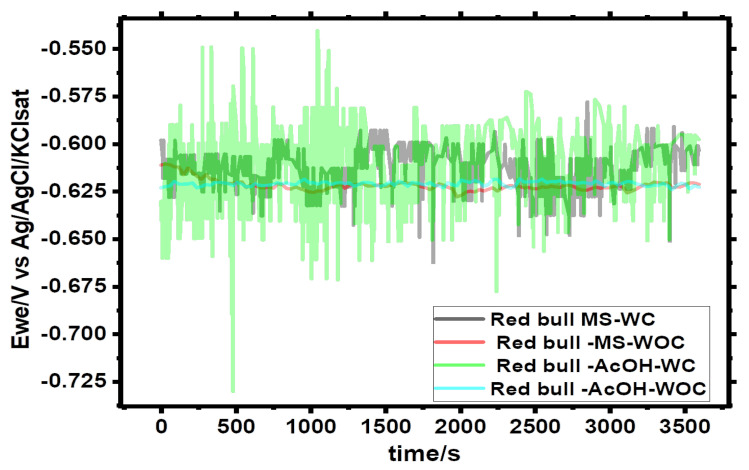
Open circuit potential of Red Bull samples with (WC) and without (WOC) an organic coating in main (MS) and acetic acid (AcOH) [0.1 M] solutions; Ewe: working electrode potential.

One-hour open circuit potential curves for Green Cola and Red Bull can samples with (WC) and without (WOC) organic coating are shown in Figure 1 and Figure 2, respectively.

Under the premise that the film is porous, the open circuit potential values for Green Cola and Red Bull samples in the presence of organic coating (i.e., as made) indicate fluctuating and unstable values during the immersion time for both samples. However, more stable and accurate open circuit potential values were obtained for uncoated cans, as seen in Figure 1 and Figure 2. The averages of the corrosion potentials after nearly one hour converge toward −0.625 V/SCE for all samples.

#### 3.1.2. Electrochemical Impedance Spectroscopy

Because of its adaptability and precision, electrochemical impedance spectroscopy (EIS) is frequently used in corrosion studies to examine the corrosion performance of coated metals [17]. EIS measurements were conducted to examine the degradation of the internal organic coating in main (MS) and acetic acid (AcOH) solutions.

**Figure 3:**
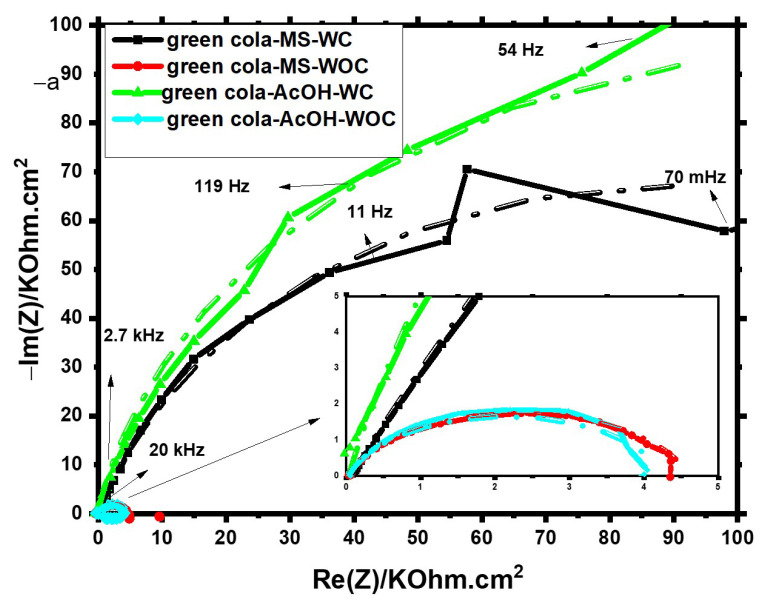
The Nyquist diagram for the Green Cola samples in main and acetic acid [0.1 M] solutions with (WC) and without (WOC) an organic coating; dash dot lines represent fitting according to Figure 5.

**Figure 4:**
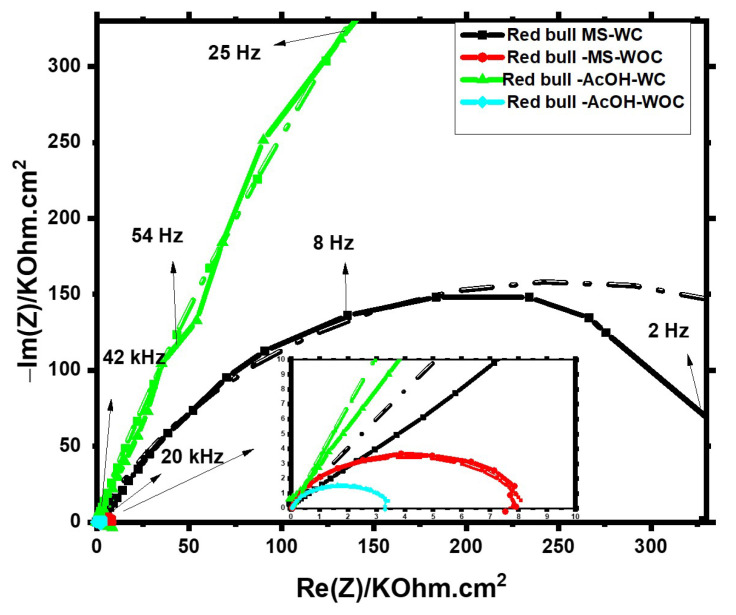
The Nyquist diagram for the Red Bull samples in main and acetic acid [0.1 M] solutions with (WC) and without (WOC) an organic coating; dash dot lines represent fitting according to Figure 5.

Figure 3 and Figure 4 represent the Nyquist diagrams used for Green Cola and Red Bull samples, respectively. Overall, after removing the internal organic coating with polishing paper, resistance values in both axes dropped drastically over the supplied frequency domain, as illustrated in the Figures’ insets (see Figure 3 and Figure 4). This indicates that the internal organic coating serves a significant function from a corrosion protection point of view. 

**Figure 5:**
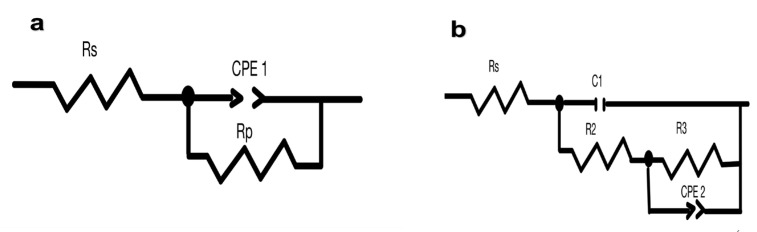
(**a**) Simplified Randles circuit for polished samples; (**b**) proposed circuit for Red Bull and Green Cola samples used to obtain impedance spectroscopy (EIS) data.

The data acquired from the impedance spectra provided in Figure 3 and Figure 4 were then fitted to an analogous electric circuit, namely a simplified Randles cell, for uncoated samples and a two-time constants circuit for coated samples, as depicted in Figure 5. Figure 5a,b are well-known models used for intact and damaged coated metals, respectively [12,18].

These cell models account for solution resistance (Rs), double-layer capacitance (CPE_1_), and (CPE_2_) for n < 1 meaning a non-ideal capacitor, where R_2_ the resistance of the coating to the transfer of ions, C_1_ ideal capacitor for coating as an organic isolator and (R_p_) and (R_3_) are charge transfer resistances of the polished aluminium metal and exposed metal through conductive paths of the coating and oxide layers, respectively. One depressed semicircle, which is indicative of a single charge-transfer, is clearly present for the uncoated samples. However, for coated samples, the diffusion process predominates at low frequencies, leading to noisy behaviour and ill-defined segments. Table 1 and Table 2 summarize the data derived from impedance spectra using the parameters listed there.

Coated samples of Green Cola and Red Bull showed double layer capacitance values significantly higher than those observed with polished samples. This signifies that more ionization occurred on the surfaces without the internal organic coating. In addition, the polarization resistance (R3) values for Green Cola and Red Bull samples with organic coating (WS) are much higher compared with Rp values without a coating (WOC), ranging from 31 to 1349 folds for Green Cola’s main and Red Bull’s AcOH solutions, respectively. An indication of smoother surfaces is a slight rise in n values for uncoated samples. According to Rp values, acetic acid addition was detrimental to uncoated samples, whereas main solutions were more corrosive for coated samples. The impedance results for Green Cola are comparable to those reported for cola-flavored soft drinks [12].

#### 3.1.3. Polarization Measurements

**Figure 6:**
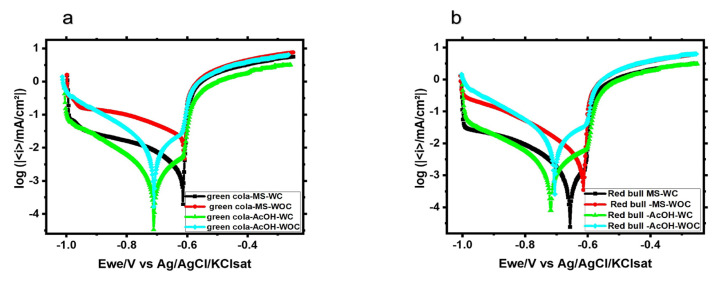
Potentiodynamic polarization curves of (**a**) Green Cola and (**b**) Red Bull samples in main and acetic acid [0.1 M] solutions with and without an internal organic coating; Ewe: working electrode potential.

Following open circuit potential measurements, potentiodynamic polarizations were conducted to evaluate the corrosion resistance of the samples under four conditions (MS-WC, MS-WOC, AcOH-WC, and AcOH-WOC). Representative Tafel plots are depicted in Figure 6. The corrosion current density and corrosion rate values were extracted from each polarization curve and are summarized in Table 3.

The samples from Green Cola examined in the main solution of the soft drink (MS-WOC) had the highest corrosion current density with an i_corr_ of 41.3 µAcm^−2^, whereas the same samples in the AcOH-WOC solution had a lower corrosion current density with an i_corr_ of 7.5 µAcm^−2^, as depicted in Figure 6a. This indicates that the Green Cola soft drink’s main solution is more corrosive than acetic acid alone. The uncoated samples of Red Bull (4.3 mpy) showed a lower corrosion current density compared with Green Cola (17.7 mpy) in main solutions, and the coated samples had a comparable contrast. Overall, the presence of the internal organic coating reduced the corrosion rate by 80 to 99%. Accordingly, organic-coated Green Cola and Red Bull samples demonstrated greater corrosion resistance, while samples without the organic coating showed an increase in current and decreased corrosion resistance (Table 3). The samples studied in acetic acid displayed a negative shift in the corrosion potential for both types. This was probably related to an increase in the acidity close to the surface. Therefore, this resulted in an increase in H^+^ ion concentration and cathodic current. E_corr_ values were almost identical regardless of the presence or absence of the organic coating owing to porous or damaged coating. Samples of Red Bull showed similar results, but to a lesser extent. 

#### 3.1.4. Cyclic Polarization

**Figure 7:**
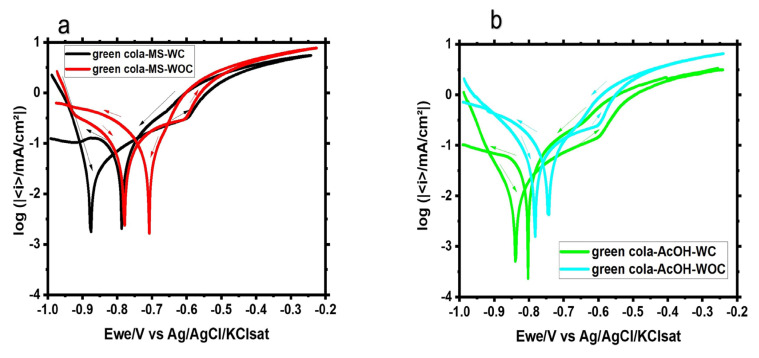
Cyclic potentiodynamic curves for the Green Cola samples with (WC) and without (WOC) an organic coating studied in (**a**) the main solution of the soft drink and (**b**) acetic acid; Ewe: working electrode potential.

**Figure 8:**
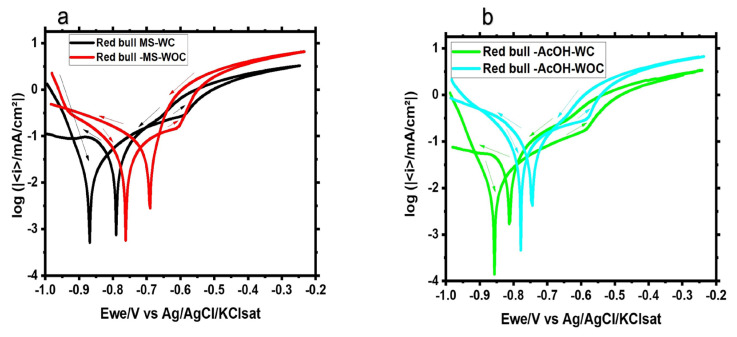
Cyclic potentiodynamic curves for the Red Bull samples with (WC) and without (WOC) an organic coating studied in (**a**) the main solution of the soft drink and (**b**) acetic acid; Ewe: working electrode potential.

The polarization was carried out with and without the organic coating, as shown in Figure 7 and Figure 8. Several observations can be noticed. At both electrolytes, organic coated samples exhibit more negative corrosion potential, although the difference in the potential (ΔE) between corrosion potential (E_corr_) and re-passivation potential (E_rep_) remains similar. E_rep_ lies on nobler voltages than E_corr_ for all samples, indicating aluminum oxide formation, but to a lesser extent for samples in acetic acid solutions owing to the higher acidity of the solution. Moreover, the measured current density of samples without an organic coating is higher than that of samples with an organic coating. In addition, the hysteresis loop in the presence of the organic coating is wider, thereby implying enhanced corrosion protection.

### 3.2. Scanning Electron Microscopy (SEM)

**Figure 9:**
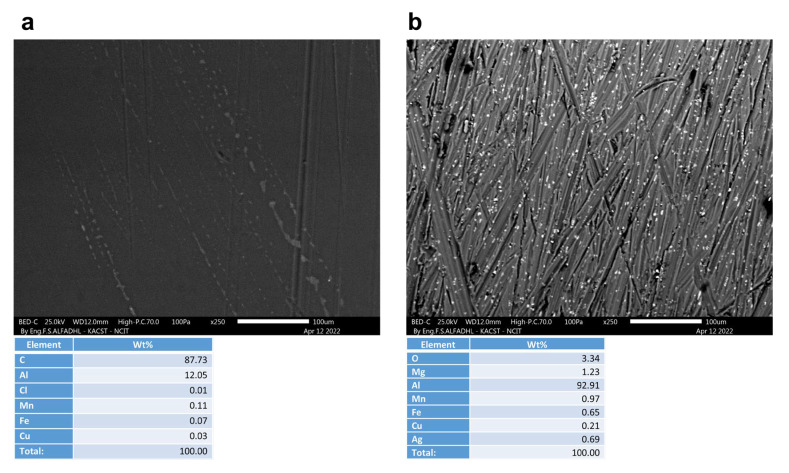
SEM and EDS analysis of untested, blank Red Bull samples (**a**) with an organic coating (WC) and (**b**) without an organic coating (WOC).

**Figure 10:**
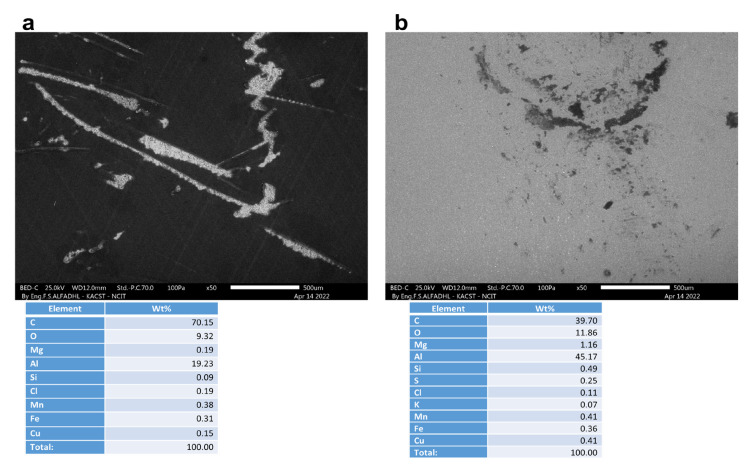
SEM and EDS analysis of Green Cola samples after corrosion tests under different conditions: (**a**) main solution in the presence of an organic coating (WC) and (**b**) without an organic coating (WOC).

**Figure 11:**
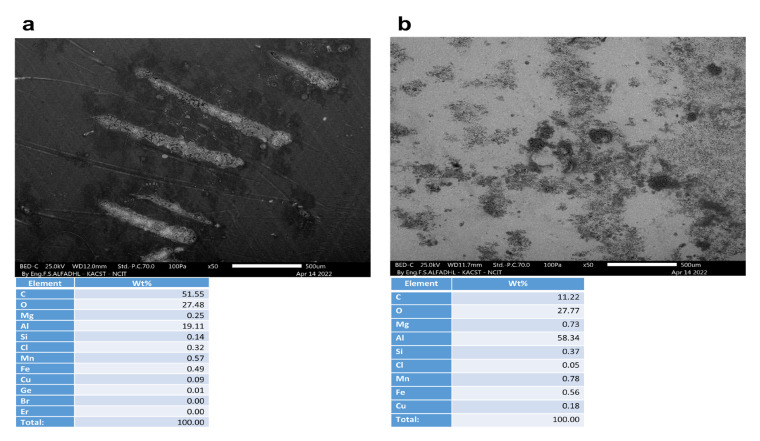
SEM and EDS analysis of Green Cola samples after corrosion tests under different conditions: (**a**) in acetic acid in the presence of an organic coating (WC) and (**b**) without an organic coating (WOC).

**Figure 12:**
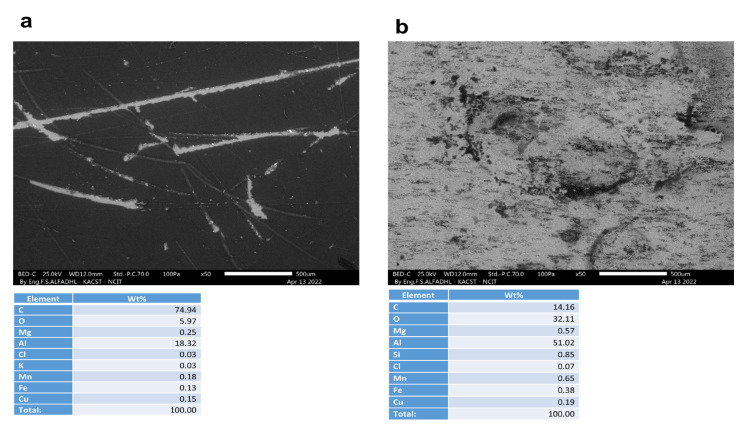
SEM and EDS analysis of Red Bull samples after corrosion tests under different conditions: (**a**) main solution in the presence of an organic coating (WC) and (**b**) without an organic coating (WOC).

**Figure 13:**
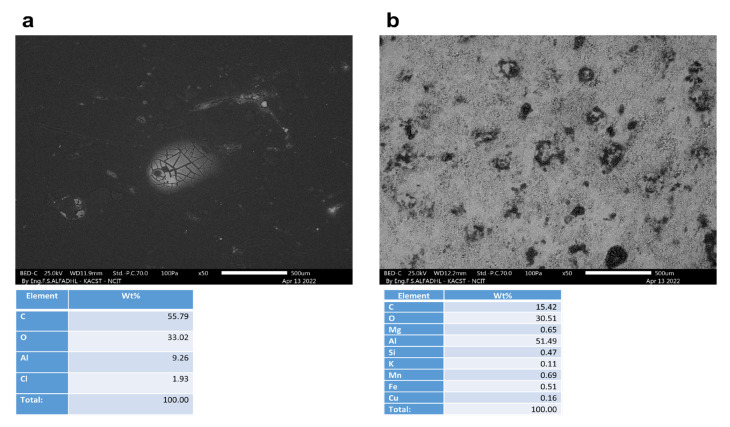
SEM and EDS analysis of Red Bull samples after corrosion tests under different conditions: (**a**) in acetic acid in the presence of an organic coating (WC) and (**b**) without an organic coating (WOC).

A non-corroded contact blank sample, with and without a coating, is shown in Figure 9. As depicted in Figure 10, Figure 11, Figure 12 and Figure 13, the morphology of the samples was examined following corrosion tests in both main (MS) and acetic acid [0.1 M] solutions. 

Corrosion areas were clearly visible for samples without an organic coating (WOC), whether examined in the main (MS) or in the acetic acid solutions for both types (i.e., Green Cola and Red Bull). As shown in (a) of Figure 10, Figure 11, Figure 12 and Figure 13, corrosion with an organic coating is more localized and limited in scope, as indicated by the grey areas, compared with the regions depicted in (b) of Figure 10, Figure 11, Figure 12 and Figure 13. From EDS analysis, the blank sample showed some aluminium content as (C = 87.7, Al = 12.1) and the polished sample in Figure 9b showed almost pure aluminium. Furthermore, EDS analysis in Figure 10 shows a still high carbon percentage, but increased aluminum for coated samples (C = 70.2, Al = 19.2, O = 9.3), whereas in the absence of the coating, carbon and oxygen also spiked, implying the formation of corrosion products (C = 39.7, Al = 45.2, O = 11.1). Similar trends are observed in Figure 11, Figure 12 and Figure 13. In addition, aluminium alloy constituents, such as mg, Cu, Mn, and Fe, are more prevalent after corrosion for coated samples, as a result of coating failure (see Figure 10, Figure 11 and Figure 12). Figure 13a illustrates a Red Bull AcOH WC sample with the most intact coating and fewest corrosion products. In accordance with Table 1’s high impedance resistance, an increase in oxygen content could indicate the formation of aluminium oxide.

### 3.3. Elemental Analysis

Analytical techniques such as inductively coupled plasma optical emission spectrometry (ICP OES) are useful for determining the concentration of components in samples [19]. ICP OES was performed with either Green Cola, Green Cola with KCl, or acetic acid with KCl solutions, whereby, KCl was added to improve conductivity. Leakage of aluminium was examined using ICP OES in this investigation. After an electrochemical corrosion test on Green Cola, the aluminium content leached into the main solution as a corrosion electrolyte was 0.75 ppm, which is above the accepted contamination level, 0.2 mg/L, stipulated by the Agency for Toxic Substances and Disease Registry (ATSDR) [20]. This content increased significantly to 20.70 ppm after using supporting electrolyte (KCl). Furthermore, the amount of aluminium that leached from the Green Cola sample into acetic acid as a corrosion electrolyte was 13.20 ppm. Accordingly, solution conductivity and severity of content plays a significant role in soft drink can corrosion. The corrosion results in Table 3 and EDS analysis in Figure 10a are consistent with higher aluminium concentrations in the Green Cola main solution as determined by the (ICP OES) technique. 

### 3.4. X-ray Diffraction Study

**Figure 14:**
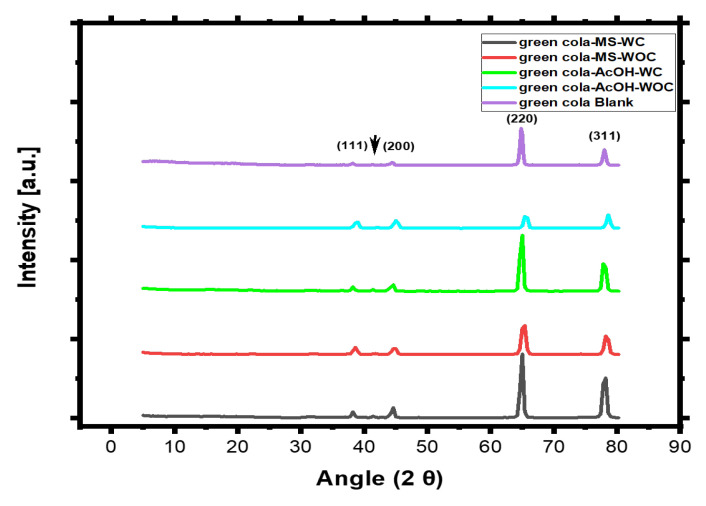
XRD patterns of Green Cola samples after corrosion tests with (WC) and without (WOC) an organic coating studied in the main solution of the soft drink and in acetic acid.

Figure 14 represents the XRD of aluminium cans for Green Cola after corrosion in main and acetic acid solutions with and without a coating and a blank sample. All five figures display pure aluminium peaks, and a small peak appears for corroded samples as a result of impurities such as manganese, magnesium, and iron, which is highlighted by the arrow in the Figure [21]. The aluminium peaks at 2θ = 39.3, 45.5, 65.8, and 78.8° in Figure 14 correspond to the pure aluminium profile in the literature (JCPDS number 89-4037) [22]. For the indexed peaks, there are three phases: boehmite (AlOOH) for 2θ = 39.3 and 65.8°, face centred cubic aluminium for 2θ = 65.8 and 78.8°, and cubic γ-Al2O3 for 2θ = 45.5° [22]. Figure 14 clearly indicates that uncoated samples had lower crystallinity as the intensity of the (220) and (311) planes decreased owing to the corrosion of unprotected aluminium metal. In contrast, the uncoated samples’ planes (111) and (200) peaks widened and remained the same, respectively, compared with the coated samples, which could be related to corrosion products’ formation following aluminium oxidation. Green Cola coated samples showed an out of plane shift to lower angles because of the coating, which might indicate tensile stress of the sample reducing fault probabilities and strains [23]. The blank coated sample displayed lower crystallinity than the corroded ones, which may have been a result of the coating thickness thinning or failing during corrosion, allowing for the appearance of more intensified peaks. 

## 4. Conclusions

In this investigation, aluminium cans of Green Cola and Red Bull energy drinks were analyzed in two different solutions: main and 0.1 M acetic acid. The cans were examined both with and without the interior coating.

The coating provides substantial protection against corrosion in comparison with uncoated samples, as the corrosion rate was reduced by 80 to 99 percent based on Tafel plots, and the polarization resistance obtained for EIS results increased by three orders of magnitude or more. The coating protects the metal, as evidenced by the increased intensity of aluminium peaks in coated samples, as determined by XRD results. It was discovered, despite the fact that the coating lessens the corrosion caused by the Green Cola primary solution, that the solution is still extremely corrosive. 

Similar open circuit potential values between coated and uncoated samples, two time constants impedance model involving metal participation in the reaction, aluminum leakage detection from ICP OES test, grey areas in SEM images representing corrosion products, and increasing aluminum percentage detected by EDS analysis all indicate that the coating failed to fully prevent aluminum from contaminating the drinks.

## Figures and Tables

**Table 1:** R1—solution resistance, C1—coating capacitance, R2—coating charge transfer resistance, R3—aluminium charge transfer resistance, Q3 CPE—double layer capacitance (constant phase element), n3—exponent; these are the parameters of electrochemical impedance for coated Green Cola and Red Bull samples.

	Sample Name	Rs (ohm cm^2^)	C1 (nFcm^−2^)	R2 (ohm cm^2^)	R3 (Kohm cm^2^)	Q3 CPE (µF.s^(n−1)^.cm^−2^)	n3	Chi-Square
1	Green Cola-MS-WC	50.5	8.9	73.1	180	0.40	0.80	0.163
2	Green Cola-AcOH WC	42.6	4.0	58.0	236	0.048	0.81	4.2
3	Red Bull MS-WC	23.7	0.21	39.1	505	0.40	0.71	5.9
4	Red Bull AcOH WC	63.2	2.2	350.2	4749	0.094	0.77	4.2

**Table 2:** R1—solution resistance, Q CPE—double layer capacitance (constant phase element), n—exponent, Rp—charge transfer resistance; these are the parameters of electrochemical impedance for uncoated Green Cola and Red Bull samples.

	Sample Name	Rs (ohm cm^2^)	Q CPE (µF.s^(n−1)^.cm^−2^)	n	Rp (Kohm cm^2^)	Chi-Square
1	Green Cola-MS-WOC	45.8	20.6	0.84	5.74	0.005
2	Green Cola-AcOH-WOC	53.2	13.8	0.84	4.52	0.62
3	Red Bull MS-WOC	54.8	17.2	0.90	8.32	0.39
4	Red Bull AcOH-WOC	48.8	10.7	0.89	3.52	1.02

**Table 3:** Polarization results summary: anodic and cathodic Tafel slope (βa and βc), corrosion potential (Ecoor), corrosion current density (icorr), and corrosion rate (r_corr)_ for both Green Cola and Red Bull at room temperature.

	Sample Name	βa (mV.dec^−1^)	-βc (mV.dec^−1^)	E_corr_ (V/Ag/AgCl/KClsat)	i_corr_ (µAcm^−2^)	r_corr_ (mpy)
1	Green Cola-MS-WC	53.6	213.4	−0.61	6.0	2.6
2	Green Cola-MS-WOC	83.1	277.6	−0.61	41.3	17.7
3	Green Cola-AcOH WC	106.3	167.5	−0.71	1.4	0.6
4	Green Cola-AcOH-WOC	109.7	159.3	−0.71	7.5	3.2
5	Red Bull MS-WC	84.0	163.7	−0.66	0.9	0.4
6	Red Bull MS-WOC	59.3	150.8	−0.61	10.0	4.3
7	Red Bull AcOH WC	109.4	177.1	−0.72	1.5	0.7
8	Red Bull AcOH-WOC	120.7	163.2	−0.71	10.6	4.5

## Data Availability

All the raw data supporting the conclusion of this paper were provided by the authors.
